# Flavones Isolated from *Scutellariae radix* Suppress *Propionibacterium Acnes*-Induced Cytokine Production *In Vitro* and *In Vivo*

**DOI:** 10.3390/molecules21010015

**Published:** 2015-12-24

**Authors:** Po-Jung Tsai, Wen-Cheng Huang, Ming-Chi Hsieh, Ping-Jyun Sung, Yueh-Hsiung Kuo, Wen-Huey Wu

**Affiliations:** 1 Department of Human Development and Family Studies, National Taiwan Normal University, Taipei 106, Taiwan; pjtsai@ntnu.edu.tw (P.-J.T.); tim810481@yahoo.com.tw (W.-C.H.); lillianhsieh11@gmail.com (M.-C.H.); 2 National Museum of Marine Biology and Aquarium, Pingtung 944, Taiwan; pjsung@nmmba.gov.tw; 3 Graduate Institute of Marine Biology, National Dong Hwa University, Pingtung 944, Taiwan; 4 Department of Chinese Pharmaceutical Sciences and Chinese Medicine Resources, China Medical University, Taichung 404, Taiwan; 5 Department of Biotechnology, Asia University, Taichung 413, Taiwan

**Keywords:** Chinese herb, *Scutellariae radix*, flavone, anti-inflammation, *Propionibacterium acnes*

## Abstract

*Scutellariae radix*, the root of *Scutellaria baicalensis*, has long been applied in traditional formulations and modern herbal medications. *Propionibacterium acnes* (*P.* *acnes*) in follicles can trigger inflammation and lead to the symptom of inflammatory acnes vulgaris. This study was aimed at evaluating the effect of *Scutellariae radix* extract and purified components isolated from it on inflammation induced by *P.* *acnes in vitro* and *in vivo*. The results showed the ethyl acetate (EA) soluble fraction from the partition of crude ethanolic extract from *Scutellariae radix* inhibited *P.* *acnes*-induced interleukin IL-8 and IL-1β production in human monocytic THP-1 cells. Seven flavones were isolated from the EA fraction by repeated chromatographies, and identified as 5,7-dihydroxy-6-methoxyflavone (**FL1**, oroxylin), 5,7-dihydroxy-8-methoxyflavone (**FL2**, wogonin), 5-hydroxy-7,8-dimethoxyflavone (**FL3**, 7-*O*-methylwogonin), 5,6′-dihydroxy-6,7,8,2′-tetramethoxy flavone (**FL4**, skullcapflavone II), 5,7,4′-trihydroxy-8-methoxyflavone (**FL5**), 5,2′,6′-trihydroxy-7,8-dimethoxyflavone (**FL6**, viscidulin II), and 5,7,2′,5′-tetrahydroxy-8,6′-dimethoxyflavone (**FL7**, ganhuangenin). They all significantly suppressed *P. acnes*-induced IL-8 and IL-1β production in THP-1 cells, and **FL2** exerted the strongest effect with half maximal inhibition (IC_50_) values of 8.7 and 4.9 μM, respectively. Concomitant intradermal injection of each of the seven flavones (20 μg) with *P. acnes* effectively attenuated *P. acnes*-induced ear swelling, and decreased the production of IL-6 and tumor necrosis factor-α in ear homogenates. Our results suggested that all the seven flavones can be potential therapeutic agents against *P. acnes*-induced skin inflammation.

## 1. Introduction

*Acne vulgaris* is one of the most common skin diseases. The pathogenesis is complex and incompletely understood, but inflammation is believed to be a key component [1]. *Propionibacterium acnes* (*P. acnes*), a Gram-positive anaerobic bacterium species, may play a major role in the initiation of the inflammatory reaction by stimulating the secretion of interleukin (IL)-18, tumor necrosis factor TNFα, IL-8, and IL-12 by monocytic cells, and eventually the development of inflammatory lesions [2,3]. IL-8 is the major inflammatory mediator and a strong chemotactic factor for neutrophils, basophils, and T cells. IL-8 has been implicated in mounting an inflammatory response in acne lesions [4]. In addition, the high levels of IL-1β were observed in human acne lesion, in mouse skin lesion induced by *P. acnes,* and in *P. acnes*-exposed human monocytes [5].

*Scutellariae radix*, the root of *Scutellaria baicalensis*, has been used as traditional Chinese medicine to treat allergic and inflammatory diseases in Japan and China. It is also often used to treat cardiovascular diseases, respiratory, and gastrointestinal infections [6]. Flavonoids, including baicalin, baicalein, wogonin, and oroxylin-A, have been identified in *Scutellariae radix* [7]. The extracts and the isolated compounds of *Scutellariae radix* are pharmacologically-active and show great potential in the treatment of inflammation, cancers, and virus-related diseases [8].

However, the bioactive components of *Scutellariae radix* have not yet been completely investigated. This study is aimed at exploring the suppressive effects of seven flavones isolated from *Scutellaria radix* on *P. acnes*-induced inflammation *in vitro* and *in vivo*, and their anti-acne potential.

## 2. Results

### 2.1. Effects of Scutellariae Radix Extracts on P. acnes-Induced IL-1β and IL-8 Production in THP-Cells

**Figure 1:**
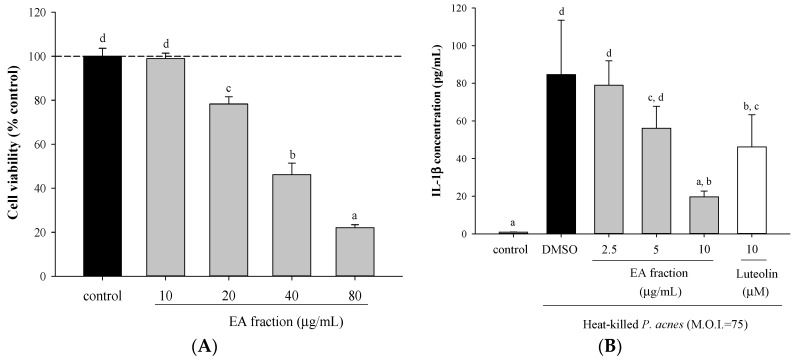

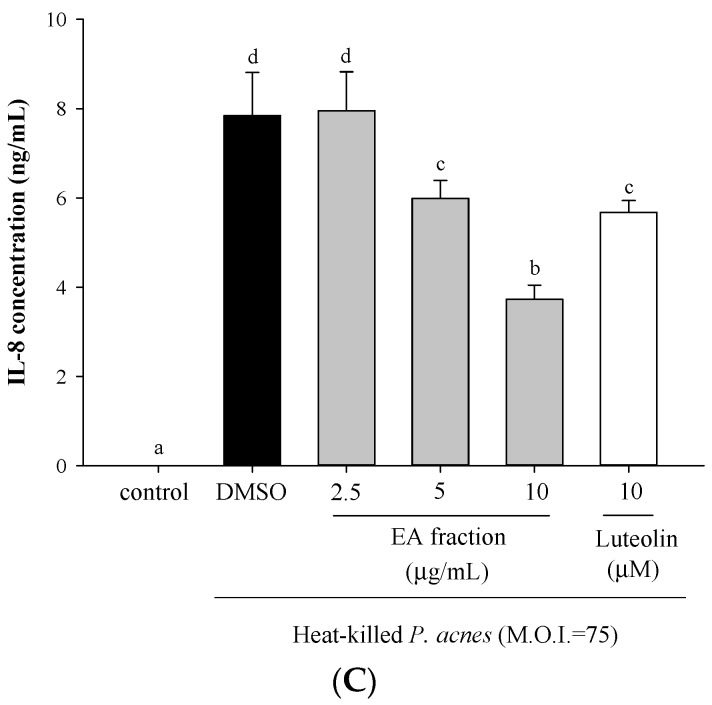
Effect of ethyl acetate (EA) fraction of ethanolic extract from *Scutellariae radix* on viability of monocytic THP-1 cells, and pro-inflammatory cytokine productions by *P. acnes*-stimulated THP-1 cells. Cell viability (**A**) was determined by MTT assay in cells incubated with vehicle control alone, or the indicated concentrations of EA fraction for 24 h. IL-1β (**B**) and IL-8 (**C**) were determined in cells co-incubated with *P. acnes* (M.O.I. = 75) and the indicated concentrations of samples for 24 h. DMSO (0.1%) was a vehicle control, luteolin was a reference control. A control experiment without *P. acnes* treatment was conducted in parallel. Each column shows the mean ± SD. Values with the same letter are not significantly different as determined by Duncan’s multiple range tests.

Ethylacetate (EA) fraction was cytotoxic to THP-1 cells when the concentrations applied were higher than 20 μg/mL (Figure 1A). So, the doses of 2.5, 5, and 10 μg/mL were used for the subsequent *in vitro* experiments. Treatment of THP-1 cells with heat-killed *P. acnes* evoked the production of IL-1β and IL-8. EA fraction of *Scutellariae radix* significantly decreased *P. acnes*-induced IL-1β and IL-8 production in a dose-dependent manner (Figure 1B,C).

The butanol fraction also inhibited IL-1β and IL-8 production by *P. acnes*-stimulated THP-1 cells, but less effectively than the EA fraction did. The butanol fraction had half maximal inhibition (IC_50_) values of 80.5 and 135.5 μg/mL for the inhibition of IL-1β and IL-8 secretion, respectively, while the EA fraction had the respective IC_50_ values of 6.6 and 5.6 μg/mL. Therefore, EA fraction was subjected to chromatography on silica gel and further purification by semi-preparative HPLC. Seven known flavones (FL1-7) were found.

### 2.2. Effects of the Seven Flavones Isolated from EA Fraction of Scutellariae Radix on P. acnes-Induced IL-8 and IL-1β Production in Human Monocytic THP-1 Cells

**Figure 2:**
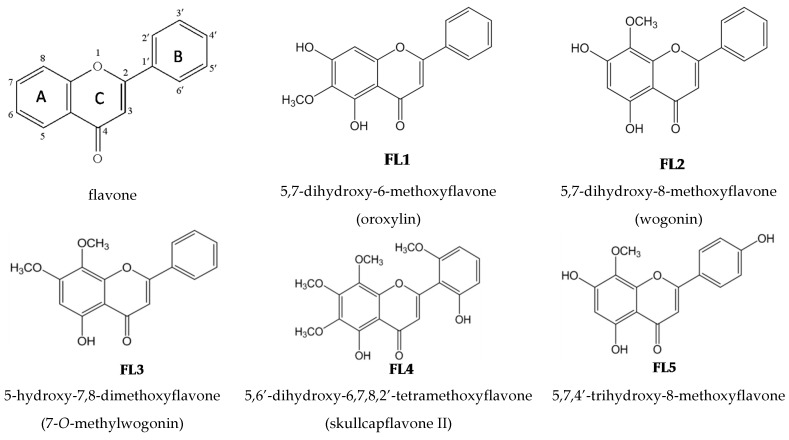

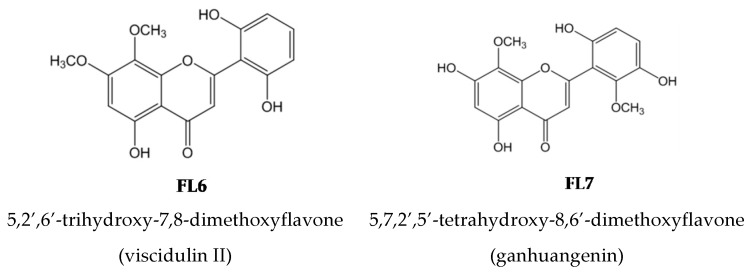
Structures of flavones isolated from *Scutellariae radix*.

Chemical names, common names, and chemical structures of the seven known flavones isolated from EA fraction of *Scutellariae radix* are shown in Figure 2.

The concentrations without apparent cytotoxicity toward THP-1 cells, assayed by MTT, were used for the subsequent experiments (Table 1). Seven flavones, at various concentrations, significantly reduced IL-8 and IL-1β levels (Table 1). The potency of the flavones was expressed as IC_50_ value. IC_50_ values of **FL4** for IL-8, and **FL1** and **FL6** for IL1β could not be precisely determined because the degree of inhibition provided by the highest test concentration was less than 50%. The rank order of potency of these flavones for IL-8 inhibition was **FL2** > **FL5** > **FL1** > (**FL4**) **FL6** > **FL3** > **FL7**; whereas for IL-1β inhibition was **FL2** > **FL4** > **FL5** > (**FL1**) > **FL3** > (**FL6**) **FL7**. Therefore, **FL2** had the most potent inhibitory effect on *P. acnes*-induced IL-1β and IL-8 production *in vitro*.

**Table 1:** Effects of flavones isolated from *Scutellariae radix* on the cell viability and *P. acnes*-induced cytokine production of THP-1 cells.

Compound	Flavone (μM)	Cell Viability (% of Control)	IL-8 Level (ng/mL)	IC_50_ for IL-8 (μM)	IL-1β Level (ng/mL)	IC_50_ for IL-1β (μM)
Control	0	93.2 ± 4.5	0.2 ± 0.2 **		0.008 ± 0.007 **	
DMSO	0	100.0 ± 2.5	52.4 ± 5.1		2.4 ± 0.2	
**FL1**	5	101.8 ± 7.8	39.2 ± 3.8 *	13.1	1.6 ± 0.2 **	NA (>15)
	10	93.3 ± 4.0	29.3 ± 5.4 **		1.5 ± 0.2 **	
	15	97.2 ± 3.0	24.1 ± 2.5 **		1.2 ± 0.1 **	
	30	64.5 ± 3.0	ND		ND	
**FL2**	5	98.6 ± 12.1	55.8 ± 8.0	8.7	1.2 ± 0.1 **	4.9
	10	112.0 ± 13.0	18.9 ± 3.5 **		0.6 ± 0.1 **	
	15	94.7 ± 2.1	5.9 ± 1.3 **		0.3 ± 0.1 **	
	30	88.4 ± 4.0	ND		ND	
**FL3**	30	99.9 ± 7.1	39.1 ± 12.0 *	55.2	1.4 ± 0.1 **	72.8
	60	101.0 ± 7.8	24.7 ± 7.4 **		1.4 ± 0.1 **	
	120	103.6 ± 4.5	15.8 ± 3.0 **		1.0 ± 0.1 **	
	240	65.6 ± 2.4	ND		ND	
**FL4**	5	102.8 ± 2.4	56.4 ± 1.9	NA (>15)	1.6 ± 0.1 **	9.1
	10	98.8 ± 3.9	39.3 ± 1.6 *		1.1 ± 0.1 **	
	15	99.6 ± 0.5	29.6 ± 2.2 **		1.0 ± 0.1 **	
	30	87.1 ± 1.8	ND		ND	
**FL5**	5	103.6 ± 2.0	67.7 ± 10.1 **	10.2	2.0 ± 0.1 *	11.3
	10	100.1 ± 2.0	32.4 ± 2.5 **		1.3 ± 0.2 **	
	15	100.8 ± 2.5	13.5 ± 0.6 **		0.8 ± 0.1 **	
	30	78.2 ± 2.8	ND		ND	
**FL6**	15	104.3 ± 5.8	41.5 ± 8.2 *	26.1	2.0 ± 0.2 **	NA (>60)
	30	101.3 ± 4.6	23.8 ± 1.5 **		1.6 ± 0.1 **	
	60	100.9 ± 4.2	20.3 ± 3.4 **		1.4 ± 0.1 **	
	120	87.5 ± 3.1	ND		ND	
**FL7**	60	106.7 ± 6.0	33.5 ± 2.3 **	124.3	1.7 ± 0.1 **	84.3
	90	110.9 ± 1.1	28.6 ± 3.0 **		1.0 ± 0.1 **	
	120	120.7 ± 1.2	26.3 ± 1.2 **		0.8 ± 0.1 **	
	240	89.3 ± 3.2	ND		ND	

IC_50_, concentrations that provide 50% inhibition; ND, not-determined; NA, not applicable. Control, cells cultured with DMSO (0.1%) alone for 24 h. DMSO, cells cultured with DMSO (as vehicle) and *P. acnes* (M.O.I. = 75) for 24 h. FL, cells cultured with the indicated concentrations of flavones and *P. acnes* (M.O.I. = 75) for 24 h. Cytokine levels were measured using ELISA kits. * *p* < 0.05, ** *p* < 0.001, as compared with DMSO.

### 2.3. Effects of the Seven Flavones Isolated from Scutellariae Radix on P. acnes-Induced Ear Edema and Cytokine Production in Mouse Ear Homogenates

**Figure 3:**
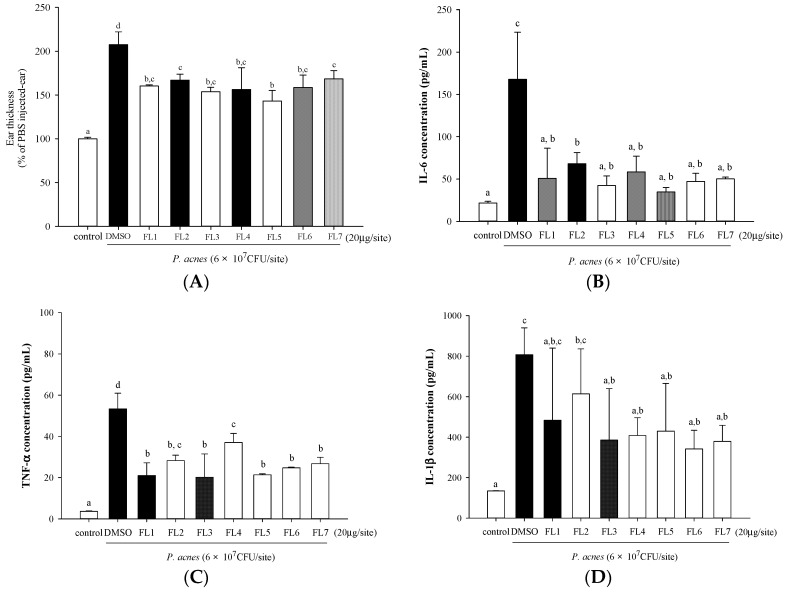
*In vivo* inhibitory effects of seven flavones isolated from *Scutellariae radix* on *P. acnes*-induced skin swelling and pro-inflammatory cytokine levels of mice ears. ICR mice were intradermally injected with PBS (as control), DMSO + *P. acnes* (as vehicle control), or flavones + *P. acnes.* Mouse ear thickness (**A**); and IL-6 (**B**); TNF-α (**C**); and IL-1β (**D**) production in mouse ear homogenates were determined. Values with the same letter are not significantly different as determined by Duncan’s multiple range tests.

Mouse ear edema was induced by intradermal injection of *P. acnes* to mouse ears. Concomitant injection of each of the seven flavones with *P. acnes* afforded suppression of *P. acnes*-induced edema as measured by ear thickness, and all the seven flavones had almost equal effects (Figure 3A). All flavones also significantly inhibited IL-6 (Figure 3B) and TNF-α (Figure 3C) production in *P. acnes-*treated mouse ears. For the suppression of IL-1β, except **FL1** and **FL2**, all the other five flavones had significant effect (Figure 3D). Our data indicated that these flavones had protective effects against *P. acnes*-induced skin inflammation.

## 3. Discussion

The pharmacological effects of the water and organic solvent extracts of *Scutellariae radix* have been extensively studied [8]. There have been over 40 flavonoids isolated from *Scutellariae radix*, with flavones and their glycosides being the most abundant [9]. Seven flavones isolated in this study existed in the forms of aglycones. **FL1** (oroxylin A) and **FL2** (wogonin) are the major flavonoids identified in *Scutellariae radix* [10]. **FL3** to **7** are minor components [8], considerably less attention has been paid to these flavones.

The anti-inflammatory effects of **FL1**, **2**, and **3** have been reported. Oroxylin A (**FL1**) inhibited LPS-induced iNOS and COX-2 gene expression by blocking NF-κB activation *in vitro* [11]. It also suppressed LPS-induced angiogenesis by down-regulation of toll-like receptor TLR-4 and the activity of mitogen-activated protein kinases (MAPK) [12]. Wogonin (**FL2**) had a very potent anti-inflammatory action *in vivo* on several animal models of inflammation, including carrageenan-induced paw edema, adjuvant-induced arthritis, 12-*O*-tetradecanoylphorbol-13-acetate (TPA)-induced skin inflammation and arachidonic acid-induced ear inflammation by oral or topical administration [13,14,15]. 7-*O*-methylwogonin (**FL3**) significantly inhibited NO and PGE_2_ release in LPS-stimulated J774A.1 macrophages [16] and effectively suppressed TNF-α, NO and macrophage inflammatory protein (MIP)-2 levels in LPS/IFN-γ-Stimulated RAW 264.7 macrophages [17]. Fewer studies investigated the biological activities of **FL4**, **5**, **6** and **7**. Skullcapflavone II (**FL4**) inhibited ovalbumin (OVA)-induced airway inflammation by reduction of Th2 cytokines, and OVA-specific IgE levels [18]. 5,7,4′-Trihydroxy-8-methoxyflavone (**FL5**) exhibited antiviral activity [19]. Viscidulin II (**FL6**) inhibited activity of adenosine 3′,5′-cyclic monophosphate phosphodiesterase [20]. Ganhuangenin (**FL7**) alleviated the type I allergic reaction by inhibiting the release of histamine and LBT4 [21]. The *in vitro* and *in vivo* *P. acnes*-induced inflammation models utilized here have never been applied to investigate the effect of *Scutellariae radix*. Therefore, our results revealed a new function of the seven flavones isolated from *Scutellariae radix*.

To compare the *in vitro* inhibitory effects of seven flavones on *P. acnes*-induced IL-8 and IL-1β production, IC_50_ values were used. We found wogonin (**FL2**) was the most potent (Table 1). **FL1**, **2**, **4**, and **5** with IC_50_ values smaller than 20 μM for both IL-1β and IL-8 were more potent than **FL3**, **6**, and **7** which had IC_50_ values larger than 50 μM. The two hydroxyl groups at C5 and C7 in **FL1**, **2**, **5** might contribute to their high potency. **FL7** also has hydroxylated C5 and C7, but showed the lowest potency probably because it has too many substituents in B ring. FL**3**, **4**, **6** with a methoxylated C7, possessing only one hydroxyl group at C5 were assumed to have lower potency. However, **FL4** was an exception, probably due to the extra methoxyl group at C6. The number of methoxyl group may also influence the potency. **FL1**, **2**, **5** have only one methoxyl group, while the less potent flavones, **FL3**, **6**, **7** have two methoxyl groups. So, fewer than two methoxyl groups might be better for potency. However, **FL4** has four methoxyl groups, but still had high potency. Nikaido, *et al.* [20] investigated the structure-activity relationship of flavones from *Scutellariae radix* for cAMP phosphodiesterase inhibition, and found when the flavones had more than five substituents, those with more methoxyl groups were more effective [20]. **FL4** and **FL7** have six, and **FL6** has five substituents. **FL4** has four methoxyl groups, while **FL6** and **7** has two. The higher potency of **FL4** than **FL6** and **7** appeared to be consistent with their hypothesis [20].

To our best knowledge, this is the first study to test the suppression effects of these flavones on *P. acnes*-induced inflammation *in vivo*. Due to the complicated cell populations involved in ear inflammation, and a fixed dose used for all the flavones, the effectiveness of each flavone cannot be completely compared as was ranked *in vitro*. However each of the seven flavones effectively reduced *P. acnes*-induced ear swelling and strongly suppressed the production of TNF-α and IL-6 in mice (Figure 3). IL-6, a pro-inflammatory and chemotactic factor [22], was measured in mice instead of IL-8 that we measured in human THP-1 cells because mice do not produce IL-8 [23].

Luteolin, a positive control in this study, is also a flavone, but without any methoxyl group. Our previous study found it inhibited *P. acnes*-induced pro-inflammatory cytokines expression by inactivating NF-κB through the suppression of mitogen-activated protein kinases (MAPK) phosphorylation [24]. Although the molecular mechanisms by which these seven flavones from *Scutellariae radix* modulate pro-inflammatory cytokine levels are not elucidated, our *in vitro* and *in vivo* results suggest each of the seven flavones has promising therapeutic potential against inflammatory *acne vulgaris*. Our results open a new aspect of the pharmacological role of these flavones.

## 4. Experimental Section

### 4.1. Isolation and Structural Elucidation

**Figure 4:**
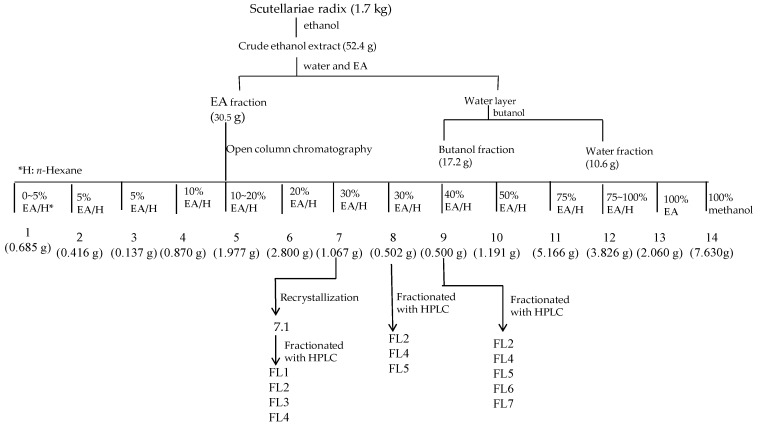
Isolation flowchart of **FL1**–**7**.

**Figure 5:**
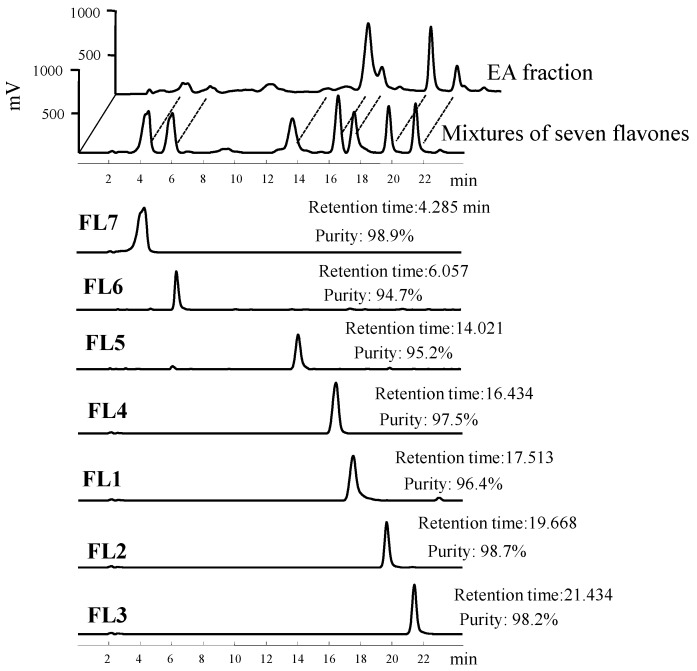
Retention time and purity analysis of **FL1**–**7** in HPLC.

*Scutellariae radix* is a Chinese herb and commercially available. We bought it from Sun Ten Pharmaceutical Co. (Taichung, Taiwan). The isolation flowchart of FL1 to 7 from *Scutellariae radix* is shown in Figure 4. Air dried pieces of *Scutellariae* *radix* (1.7 kg) were extracted twice with ethanol at room temperature (five days each time). The extract was evaporated under reduced pressure using a rotavapor to give brown residue (52.4 g). The residue was suspended in 1.5 L of H_2_O and then partitioned with 2 L of ethyl acetate (EA) twice. The water layer was partitioned with n-butanol. The combined EA soluble layer was subjected to chromatography using silica gel and further purification using a high-performance liquid chromatography (HPLC) system (Knauer, Berlin, Germany) with a Phenomenex Luna C18 column (250 × 10 mm, 5 µm) to furnish seven known flavones (Figure 4). Flavones were identified by comparing their physical and spectral data with literature values as 5,7-dihydroxy-6-methoxyflavone (**FL1**, oroxylin A) [25,26], 5,7-Dihydroxy-8-methoxyflavone (**FL2**, wogonin) [25,26,27], 5-hydroxy-7,8-dimethoxyflavone (**FL3**, 7-*O*-methylwogonin) [17], 5,6′-dihydroxy-6,7,8,2′-tetramethoxyflavone (**FL4**, skullcapflavone II) [28], 5,7,4′-trihydroxy -8-methoxyflavone (**FL5**) [29,30], 5,2′,6′-trihydroxy-7,8-dimethoxyflavone (**FL6**, viscidulin II) [31], and 5,7,2′,5′-tetrahydroxy-8,6′-dimethoxyflavone (**FL7**, ganhuangenin) [32] (Figure 2). Nuclear magnetic resonance and infrared data of the seven known compounds are available as Supplementary figures. The purity of flavones was measured by HPLC (Ecom, Prague, Czech Republic) equipped with gradient pumps (Ecom LCP 4100), a UV detector (Ecom LCD 2084) and a LiChrospher^®^ 100 RP-18E (5 µm) HPLC column (125 × 4 mm i.d., Merck Millipore, Darmstadt, Germany). The mobile phase consisting of a mixture of solvent A (water/methanol, 98:2), and solvent B (methanol/acetic acid, 98:2) was run in the following gradient mode: 0–7 min, from 50% A to 40% A with a flow rate of 0.5 mL/min; 7–12 min, from 40% A to 30% A with a flow rate of 0.3 mL/min; and 12–28 min, from 30% A to 20% A with a flow rate of 0.3 mL/min. The UV detector was at 280 nm. Chromatographic processing was done using the Peak-ABC Chromatography Data Handling System. The purities of **FL1**, **FL2**, **FL3**, **FL4**, **FL5**, **FL6**, and **FL7** were 96.4%, 98.7%, 98.2%, 97.5%, 95.2%, 94.7%, and 98.9%, respectively (Figure 5).

### 4.2. Culture of P. acnes and Preparation of Heat-Killed Bacteria

The strain of *P. acnes* (BCRC10723, isolated from facial acne) was obtained from the Bioresource Collection and Research Center (Hsinchu, Taiwan). *P. acnes* was cultured in brain heart infusion (BHI) broth (Difco, Detroit, MI, USA) with 1% glucose in an anaerobic atmosphere using BBL GasPak systems (Becton Dickinson Microbiology Systems, Cockeysville, MD, USA) at 37 °C. A spectrophotometer OD600 (Chrom Tech, Apple Valley, MN, USA) was used to determine the bacterial log phase of growth. The log-phase bacterial culture was harvested, washed thrice with phosphate-buffered saline (PBS) and re-suspended in PBS for induction of mouse ear edema. For the *in vitro* experiments, heat-killed *P. acnes* was prepared. Log-phase bacteria were washed with PBS, incubated at 100 °C for 30 min, re-suspended in RPMI medium (Gibco, Carlsbad, CA, USA) and stored at 4 °C until use.

### 4.3. Determination of the Viability of THP-1 Cells

The human monocytic THP-1 cell line (BCRC 60430) was obtained from the Bioresource Collection and Research Center (Hsinchu, Taiwan). Cells were maintained in RPMI 1640 supplemented with 10% heat-inactivated fetal bovine serum (FBS, Gibco), penicillin (100 U/mL), and streptomycin (100 μg/mL) at 37 °C in a humidified atmosphere with 5% CO_2_. To determine the cytotoxicity of tested samples, a suspension of THP-1 cells (1 × 10^6^ cells/mL) was treated with various concentrations of tested samples in 96-well culture plates for 24 h at 37 °C. Whole cell suspension was taken from each well and centrifuged for 4 min at 600 g. Supernatant was removed, and the pellet was incubated with 100 μL of MTT reagent (Sigma-Aldrich) for 3 h at 37 °C. Samples were centrifuged for 2 min at 4500 g, and supernatant was gently removed. Finally, the converted dye from the MTT reagent in cell pellets was solubilized with 500 μL of isopropanol/HCl, and then 200 μL of each sample was transferred in duplicates to a 96-well plate. The absorbance was measured using a Synergy HT multi-detection micro-plate reader (BioTek, Winooski, VT, USA) at 540 nm with 690 nm as the reference wavelength.

### 4.4. Measurement of Cytokine Production in Human Monocytic THP-1 Cells

THP-1 cells were seeded at 1 × 10^6^ cells/mL in 24-well plates with serum-free medium, and were treated with heat-killed *P. acnes* (7.5 × 10^7^ CFU/mL; multiplicity of infection (M.O.I.) = 75) alone or in combination with different concentrations of tested samples for a 24-h incubation. Cell-free supernatants were collected, and concentrations of IL-1β and IL-8 were analyzed with respective enzyme immunoassay kits (BioLegend, San Diego, CA, USA). The half maximal inhibitory concentration (IC_50_) values were estimated using a nonlinear regression algorithm (SigmaPlot 12; SPSS Inc. Chicago, IL, USA).

### 4.5. P. acnes-Induced Ear Edema and Measurement of Cytokine Levels In Vivo

Eight-week-old male ICR mice were purchased from the Animal Center of College of Medicine, National Taiwan University, Taipei, Taiwan. Animal experiments were approved by the Animal Care Committee of the National Taiwan Normal University. Mice were fed with chow diet and water *ad libitum*. To examine the anti-inflammatory effect of flavones *in vivo*, an intradermal injection model was employed [24]. In the preliminary test, 10 μL of flavones (up to 20 μg/site) was injected into mice ears. No noticeable skin irritation occurred (data not shown). Hence, flavones (20 μg/site) were used for the following experiments. Mice were randomly grouped (*n* = 5 per group). *P. acnes* (6 × 10^7^ CFU per 10 μL in PBS) was injected into the left ear of ICR mice. Right ears received an equal amount (10 μL) of PBS. Ten microliters of flavones in 5% DMSO in PBS was injected into the same location of both ears right after *P. acnes* or PBS injection. Twelve hours after bacterial injection, the increase in ear thickness was measured using a micro-caliper (Mitutoyo, Kanagawa, Japan). The increase in ear thickness of the *P. acnes*-injected ear was calculated and expressed as percentage of the PBS-injected control.

### 4.6. Measurement of Cytokine Levels In Vivo

After thickness had been measured, the ears were excised (*n* = 5 per group). The ear samples were homogenized using a BioMasher III^®^ (Nippi Inc., Tokyo, Japan) in radioimmunoprecipitation (RIPA) buffer (G-Biosciences, St. Louis, MO, USA) supplemented with 1 mM phenylmethylsulfonyl fluoride (PMSF) for 1 min on ice. The homogenates were vortexed and centrifuged at 10,000 *g* for 10 min at 4 °C. The supernatant was reserved and stored at −80 °C for the determinations of TNF-α, IL-1β, and IL-6 levels according to the manufacturer’s instruction (BioLegend).

### 4.7. Statistical Analysis

All data are presented as the mean ± standard deviation (SD). Statistical analyses were performed using the SPSS 19.0 statistical package. The data were evaluated for statistical significance with the one-way ANOVA followed by Duncan’s multiple range tests. A *p* value of < 0.05 was considered statistically significant.

## Supplementary Materials

Supplementary materials can be accessed at: http://www.mdpi.com/1420-3049/21/1/15/s1.
