# Supplementary material for: Flavones Isolated from Scutellariae radix Suppress Propionibacterium Acnes-Induced Cytokine Production In Vitro and In Vivo
Source: Molecules. 2015 Dec 24;21(1):15. doi: 10.3390/molecules21010015 (PMC6273464; doi:10.3390/molecules21010015)
Supplement: Supplementary file 1 [file molecules-21-00015-s001.pdf]

Molecules **2016**, *21*, 15; doi:10.3390/molecules21010015

Supplementary Materials: Flavones Isolated from *Scutellariae radix* Suppress *Propionibacterium acnes*-Induced Cytokine Production *In Vitro* and *In Vivo*

Po-Jung Tsai [1], Wen-Cheng Huang [1], Ming-Chi Hsieh [1], Ping-Jyun Sung [2,3], Yueh-Hsiung Kuo [4,5,*] and Wen-Huey Wu [1,*]

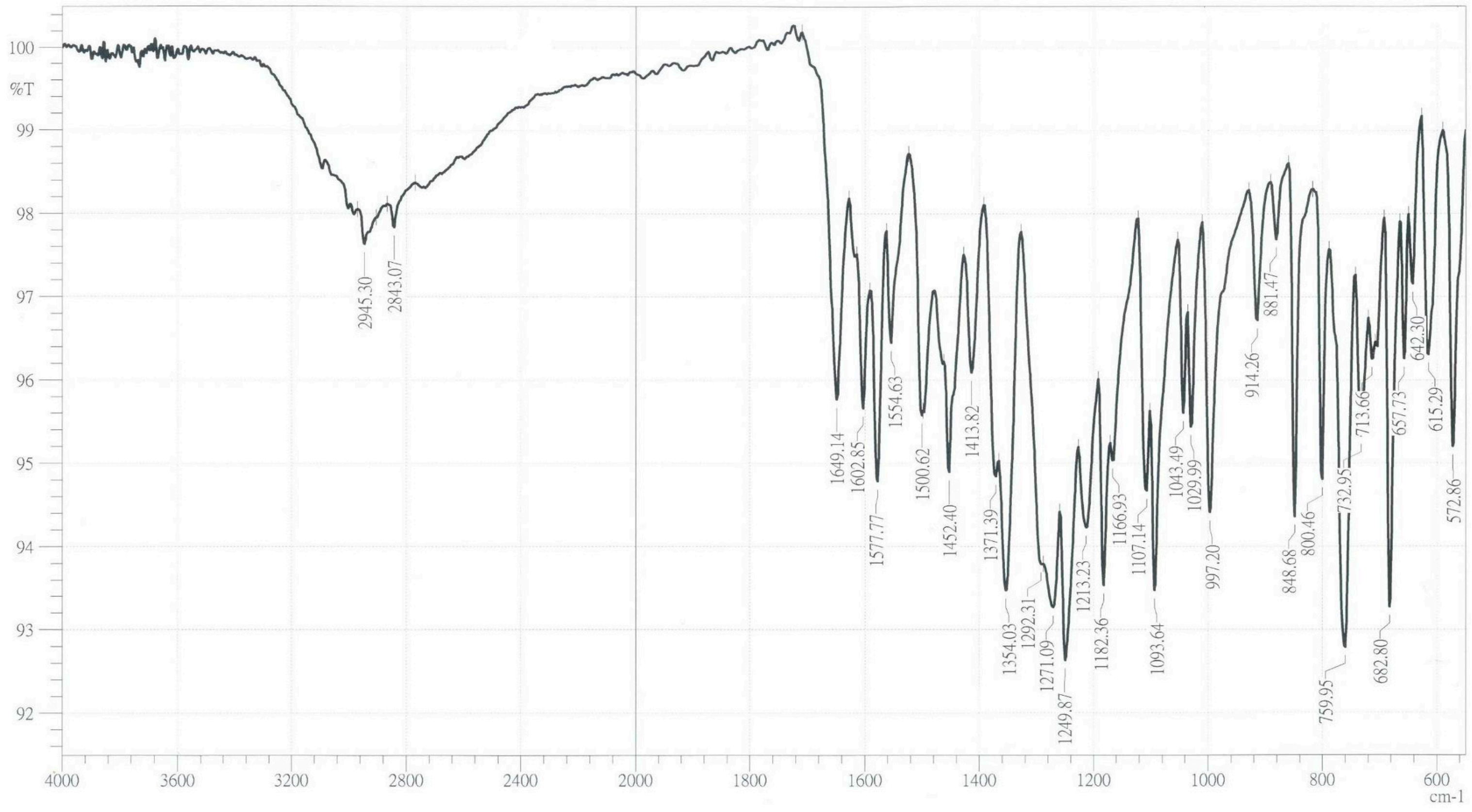

Figure S1. NMR spectrum of **FL1**.

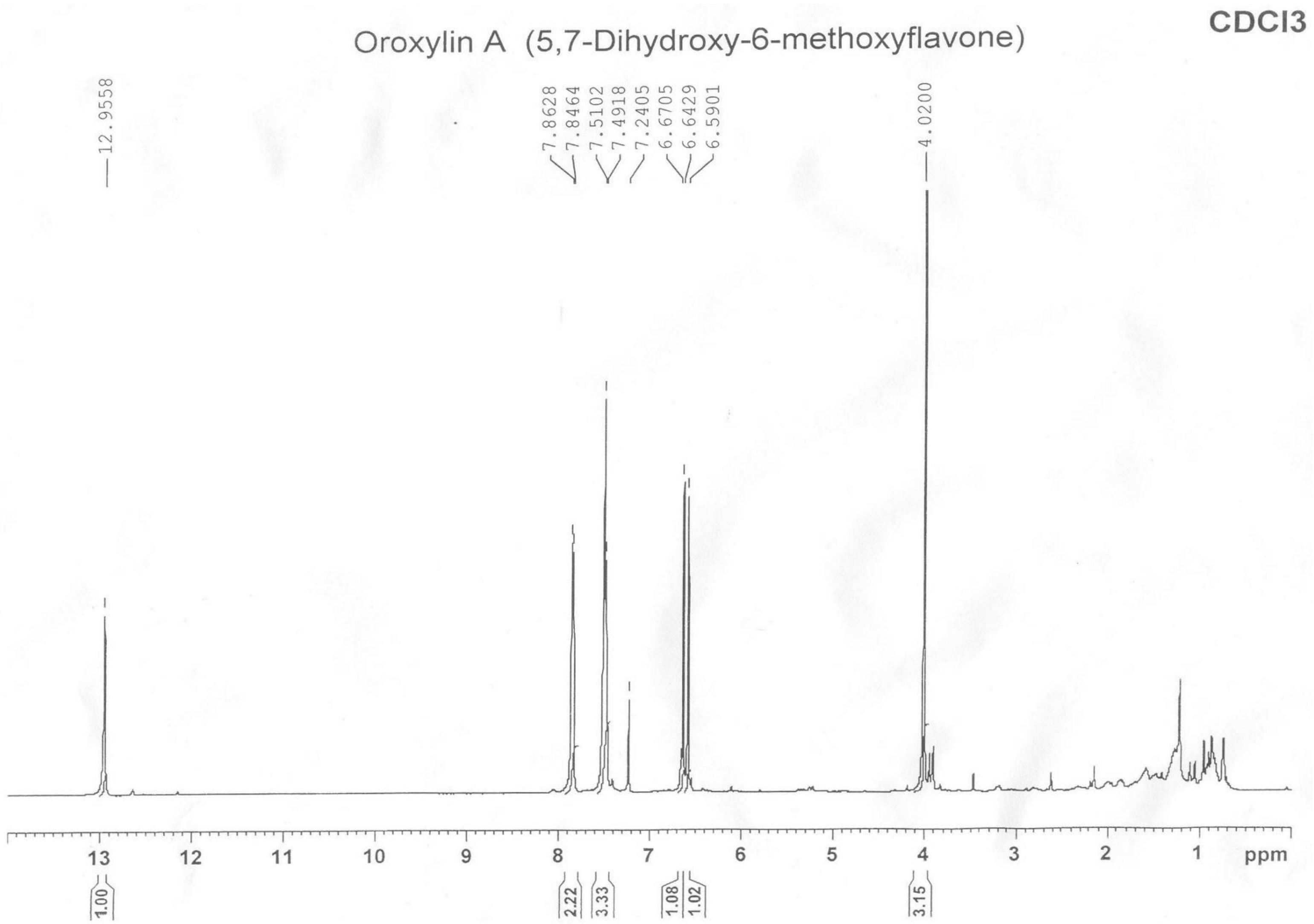

Figure S2. IR spectrum of **FL1**.

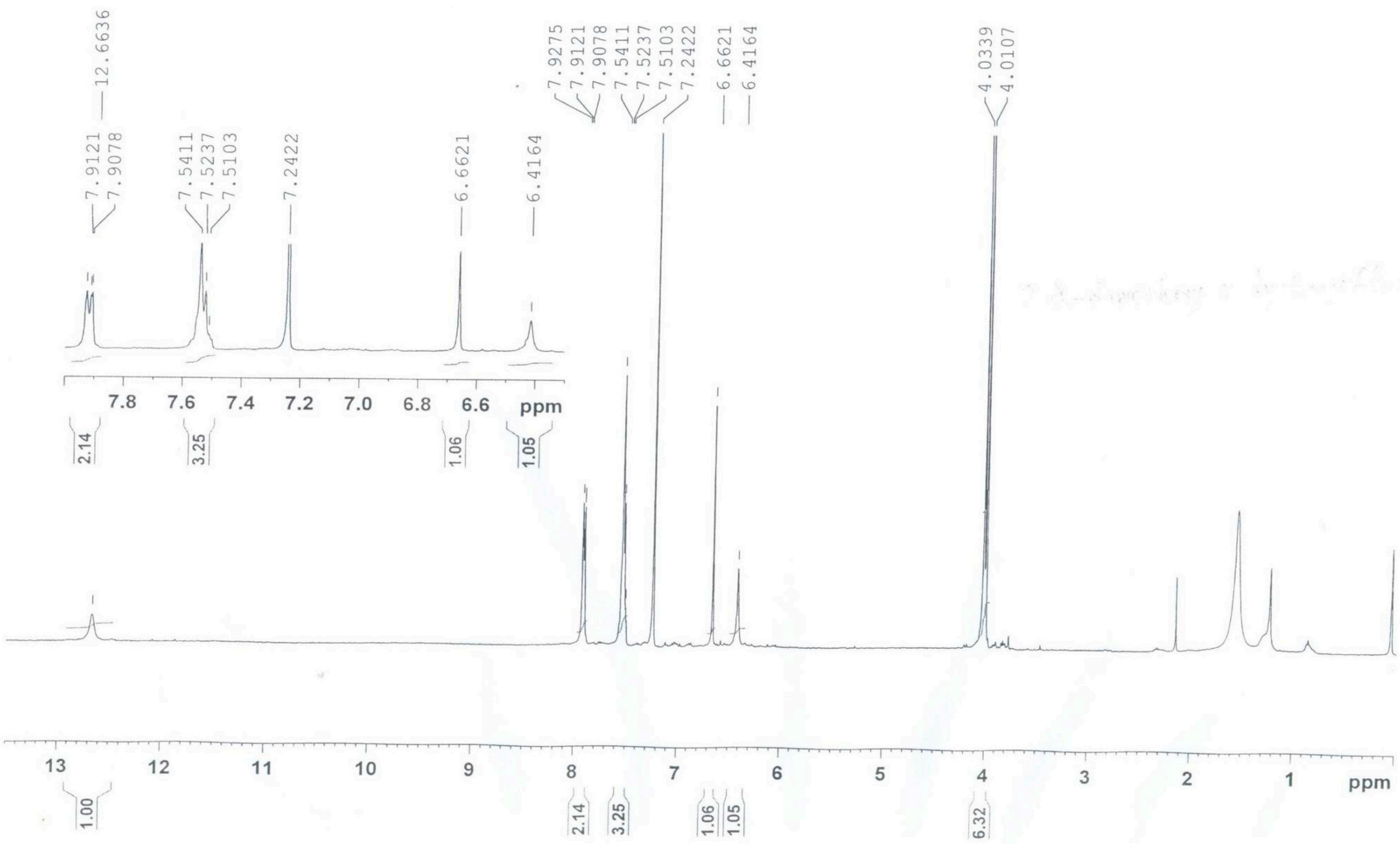

Figure S3. NMR spectrum of **FL2**.

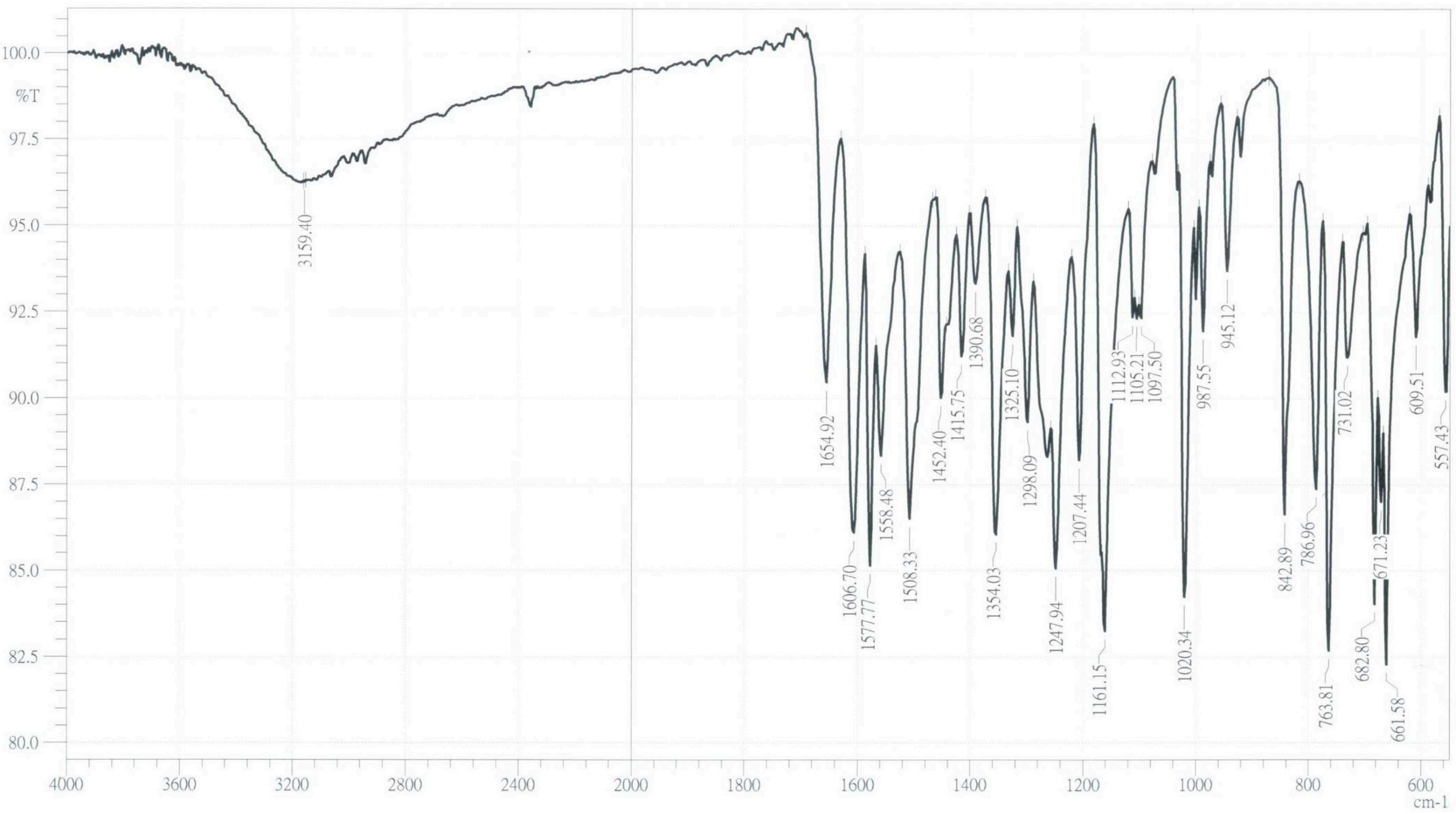

Figure S4. IR spectrum of **FL2**.

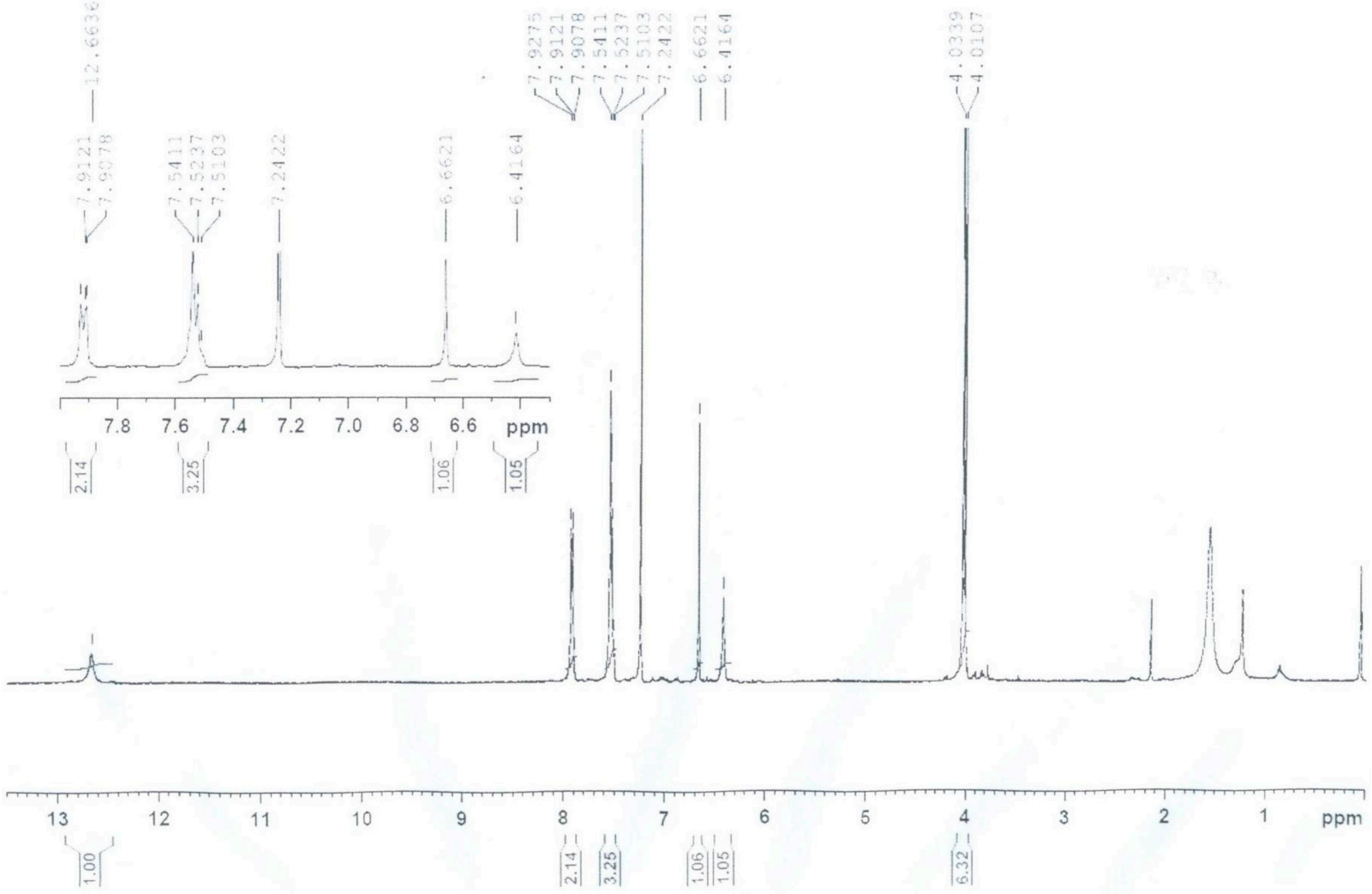

Figure S5. NMR spectrum of **FL3**.

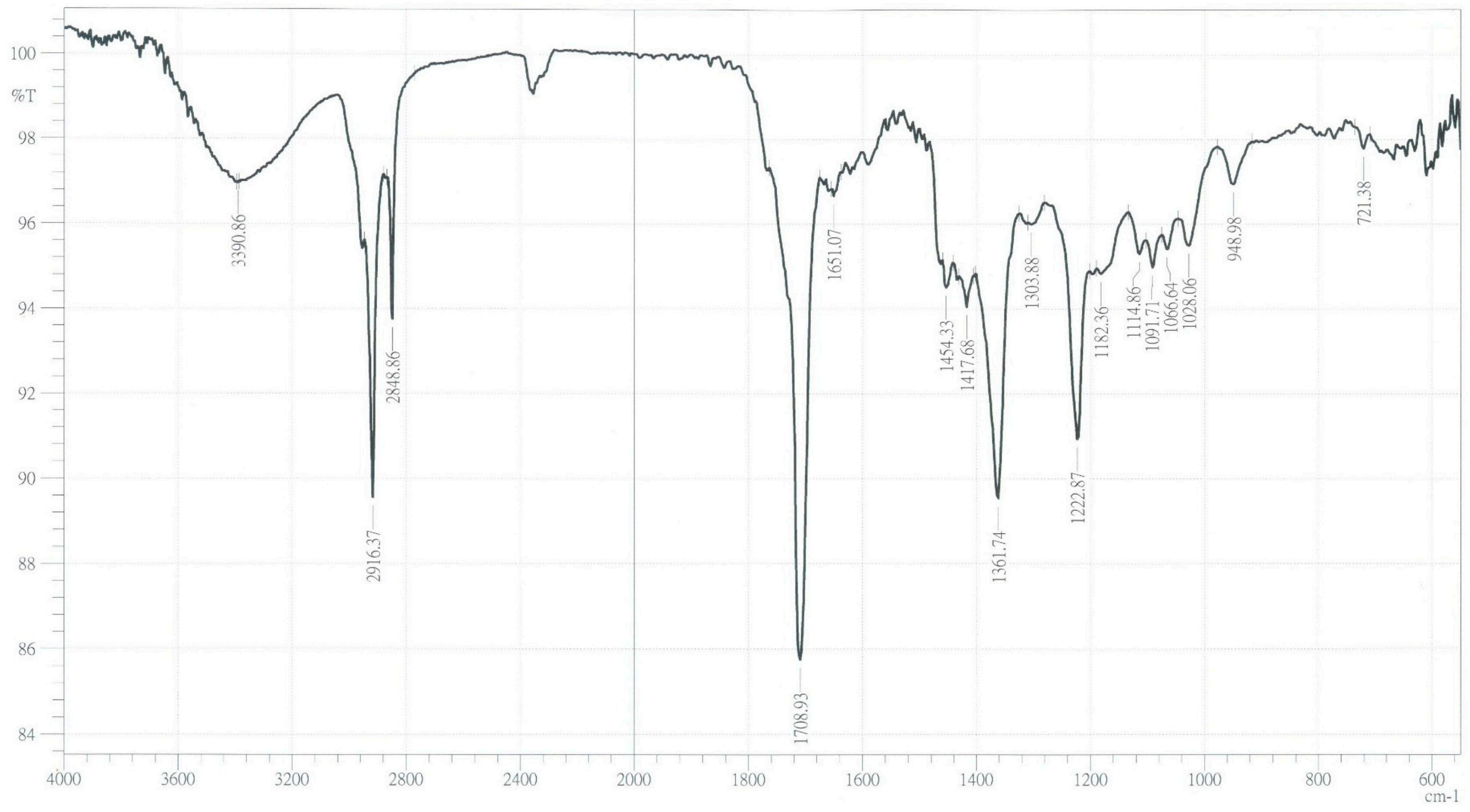

Figure S6. IR spectrum of **FL3**.

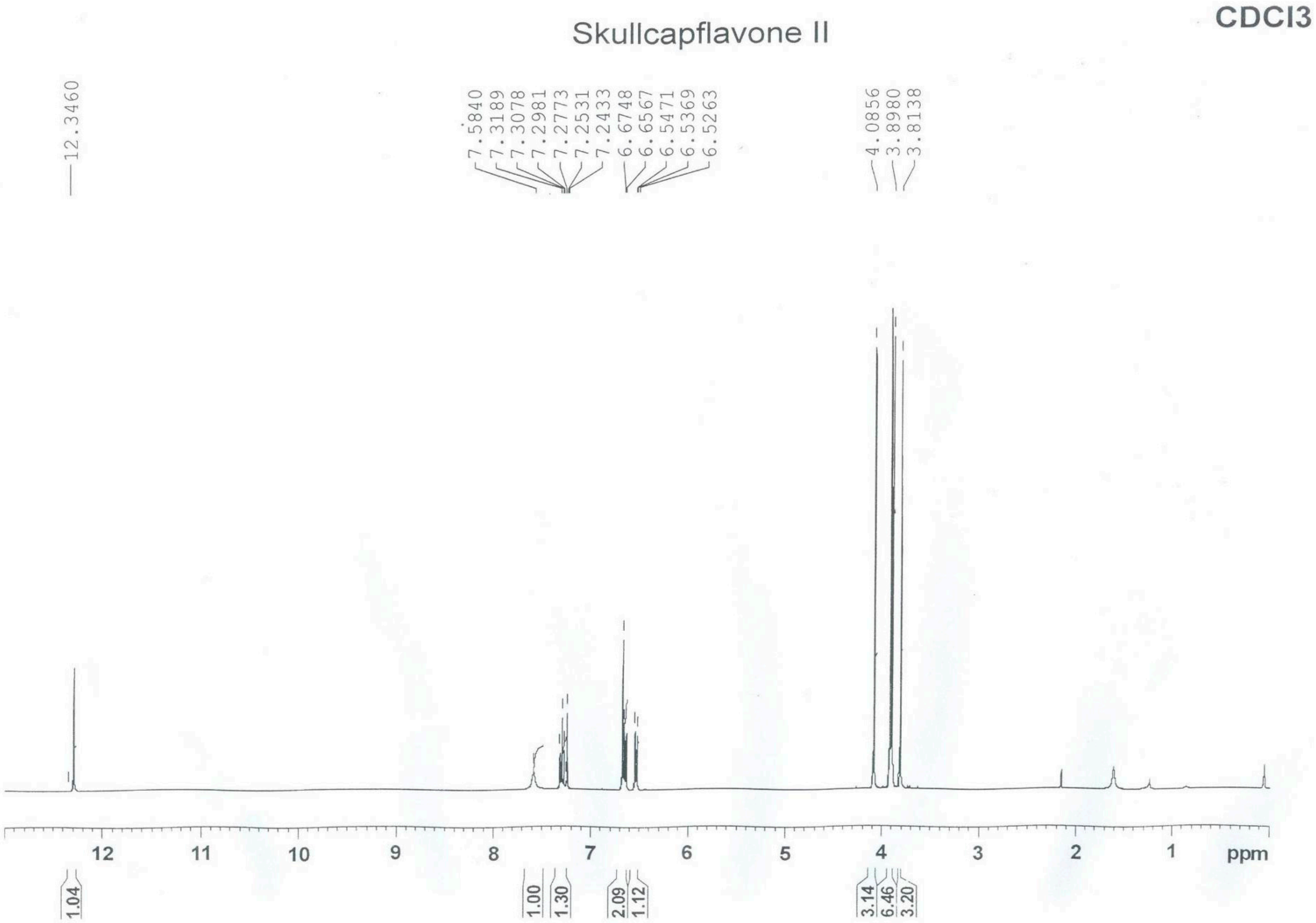

Figure S7. NMR spectrum of **FL4**.

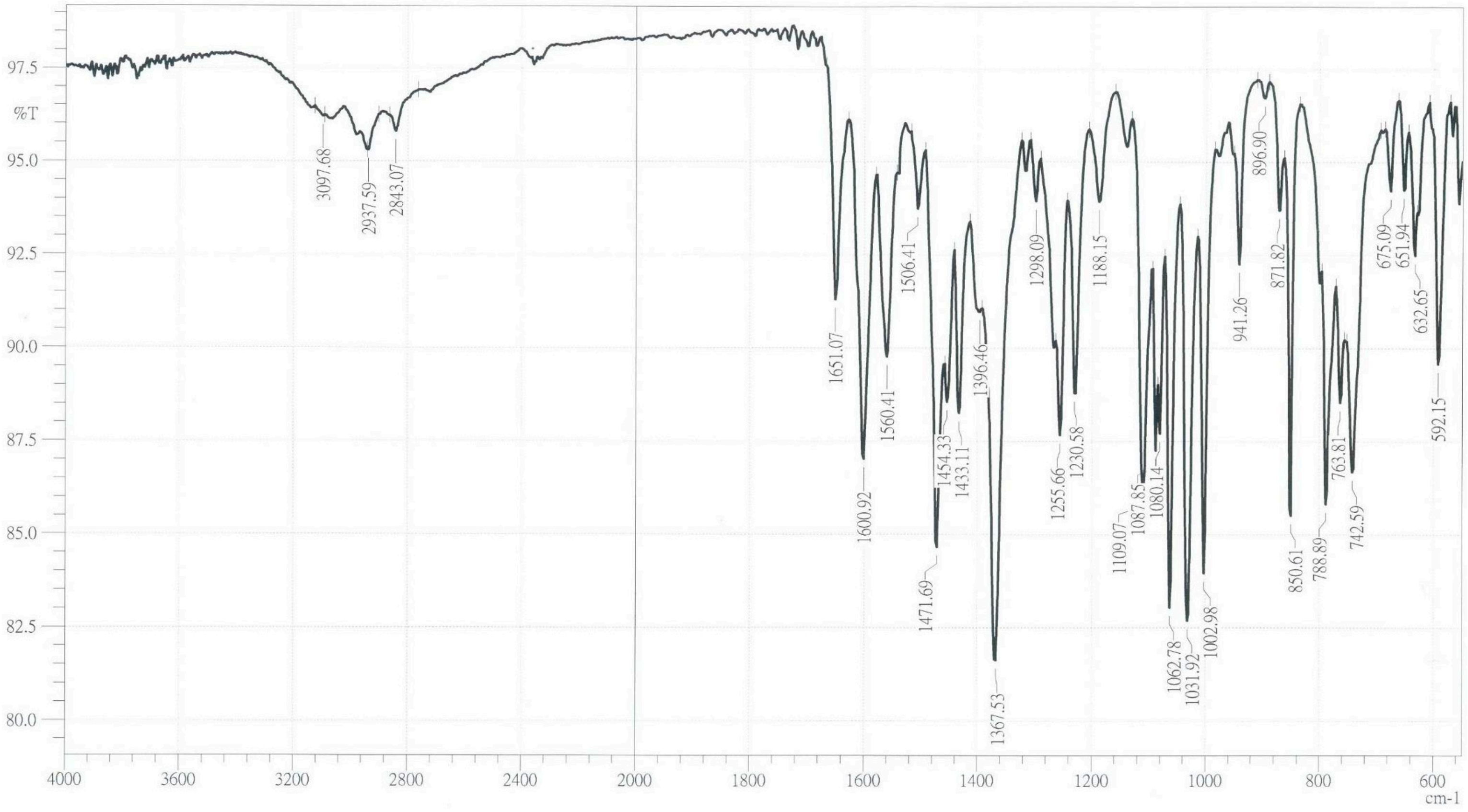

Figure S8. IR spectrum of **FL4**.

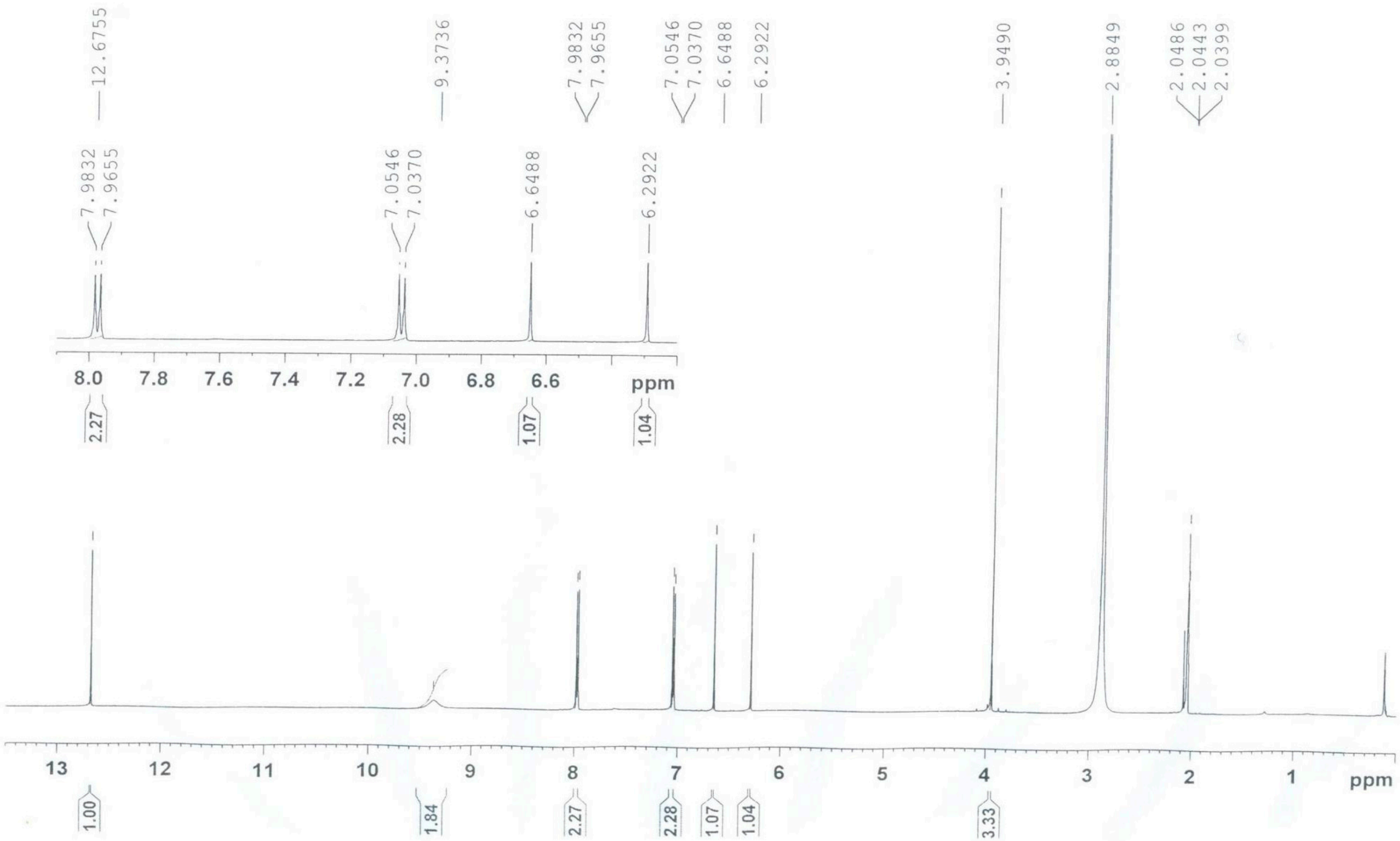

Figure S9. NMR spectrum of **FL5**.

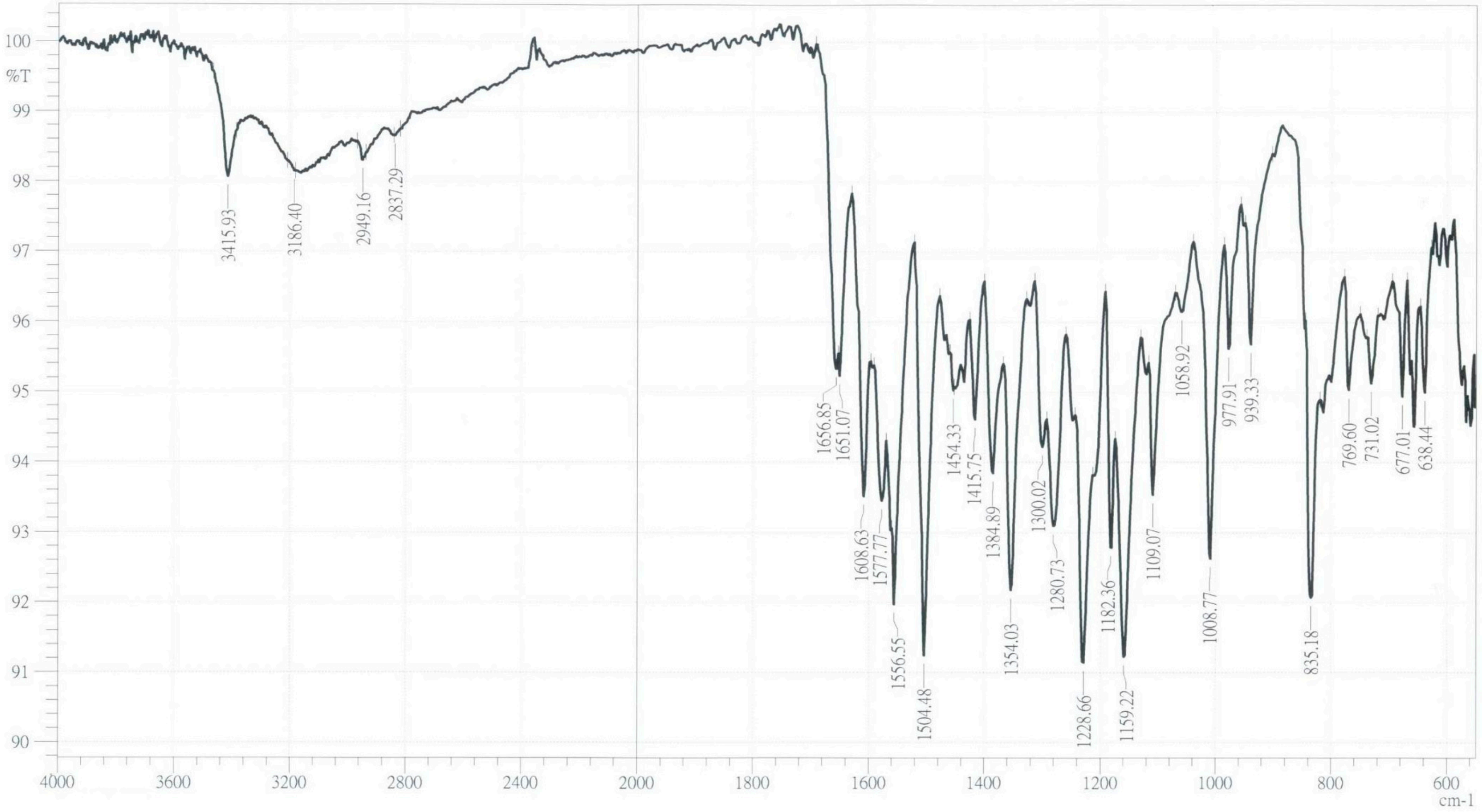

Figure S10. IR spectrum of **FL5**.

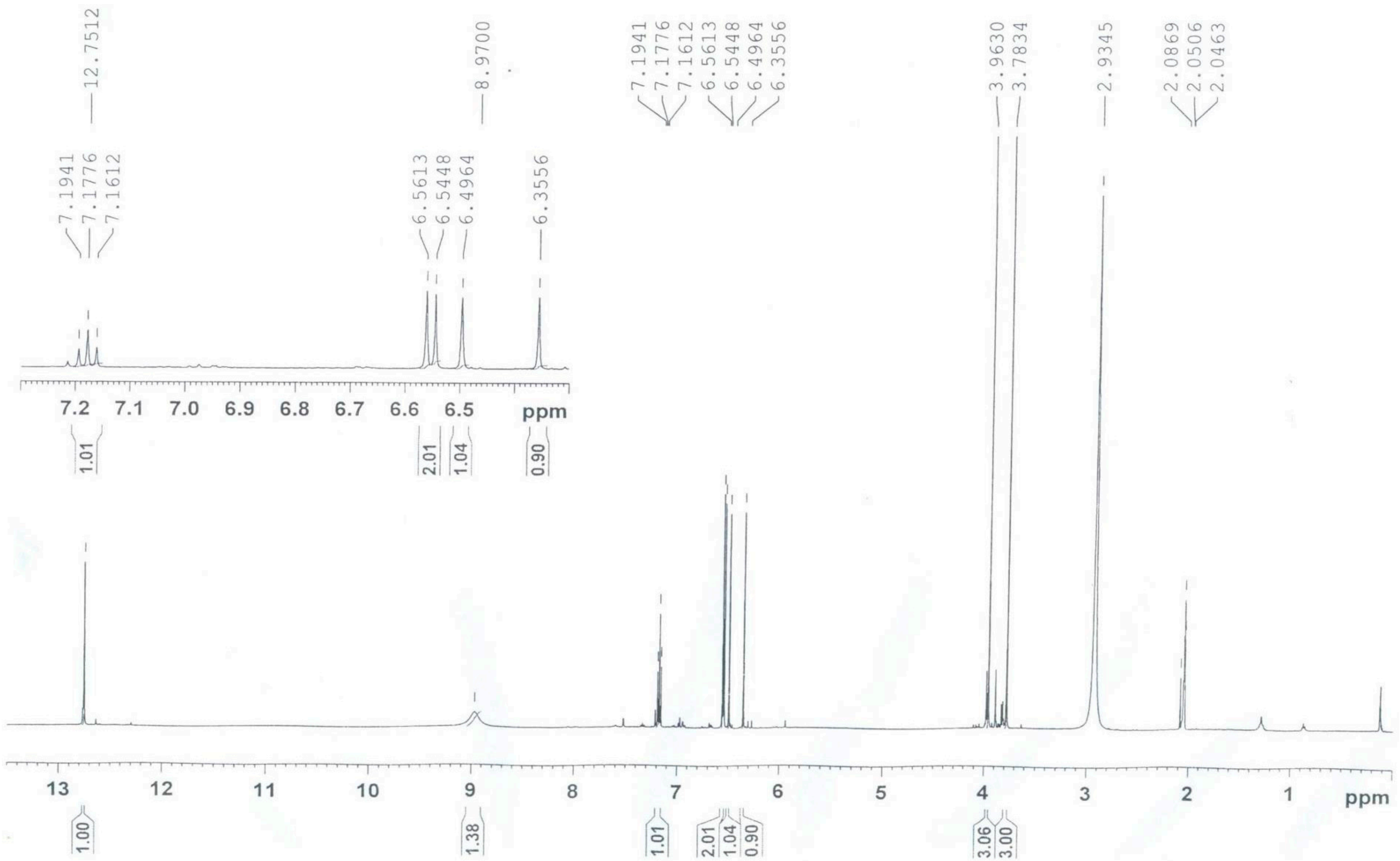

Figure S11. NMR spectrum of **FL6**.

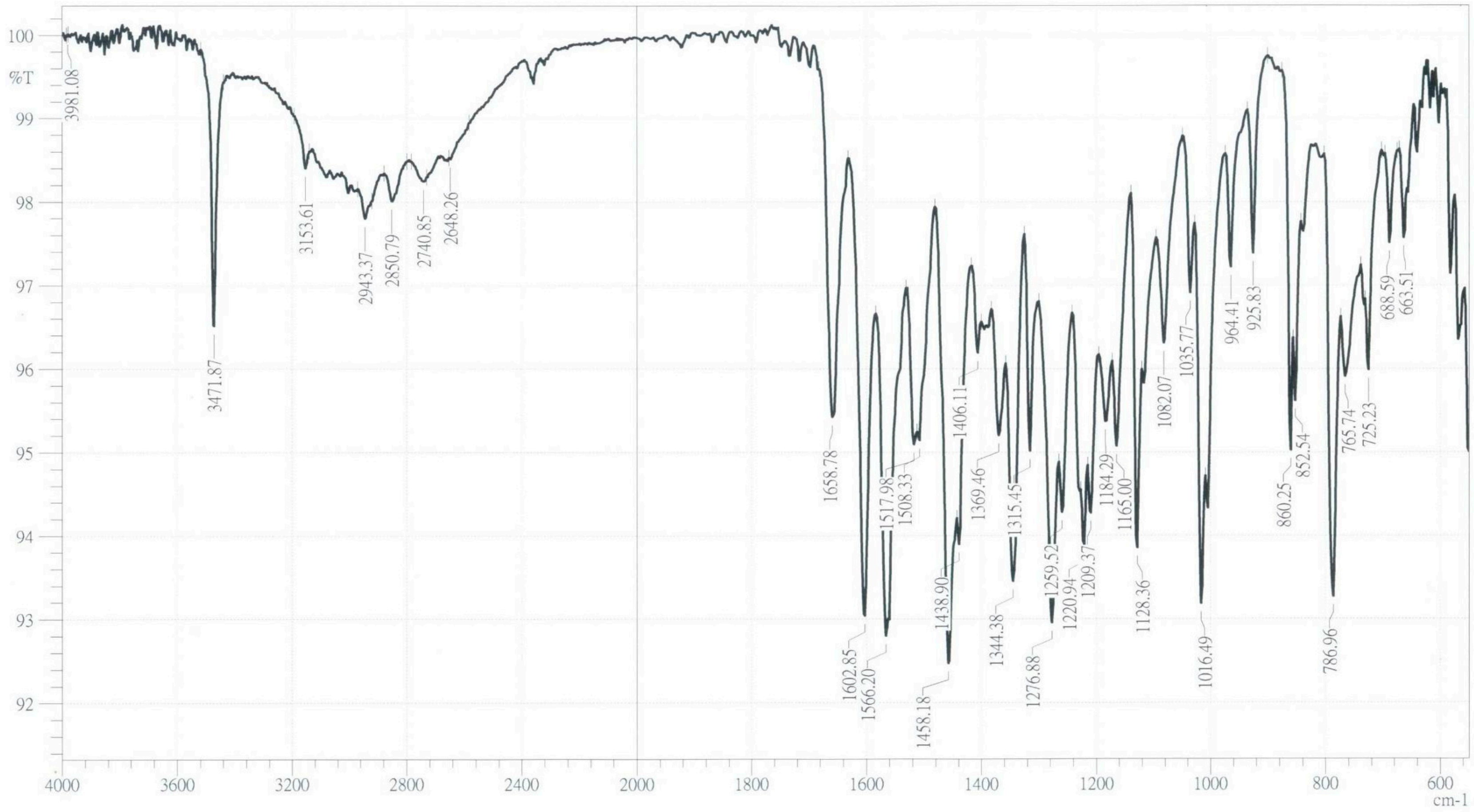

Figure S12. IR spectrum of **FL6**.

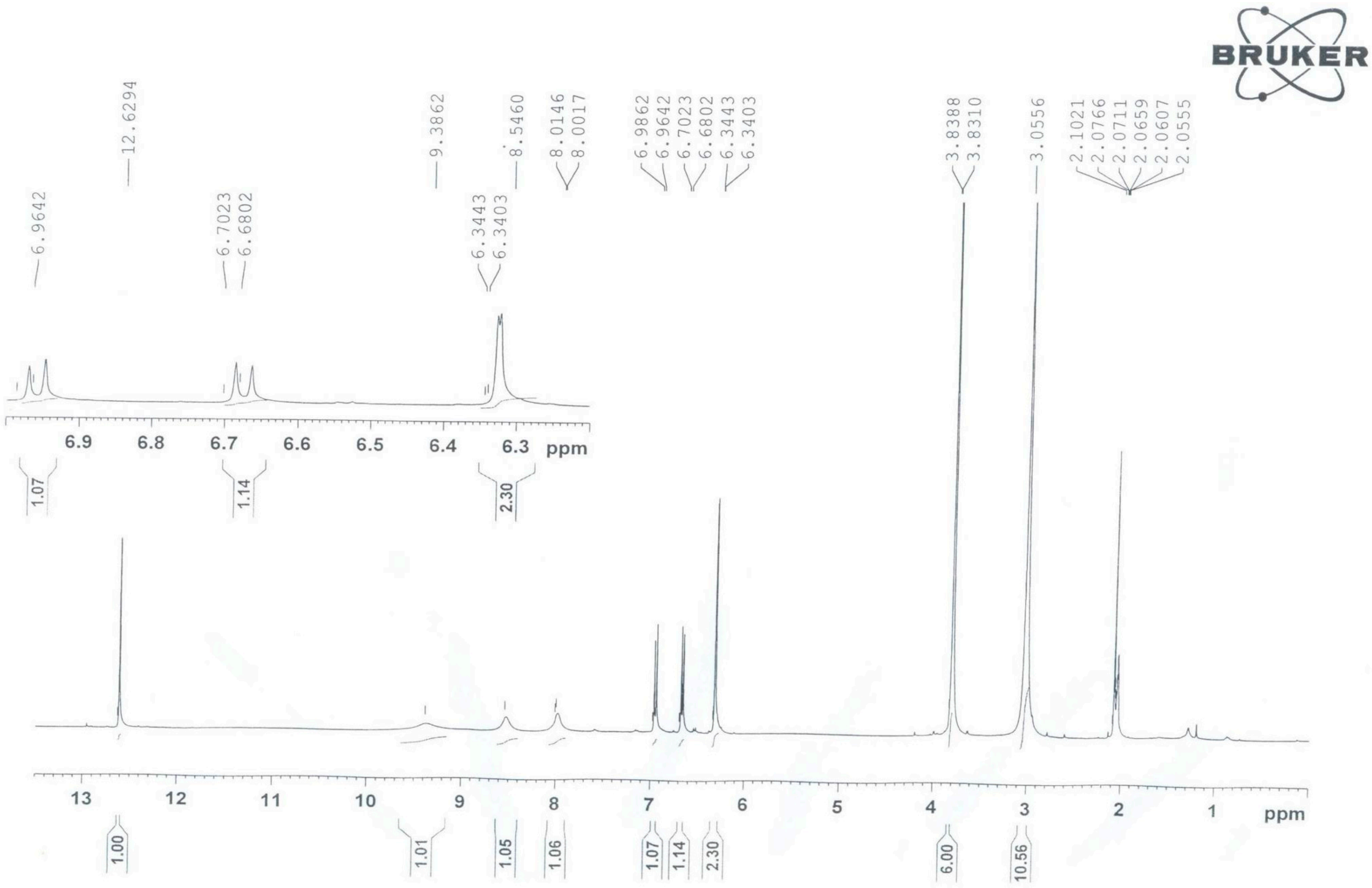

Figure S13. NMR spectrum of **FL7**.

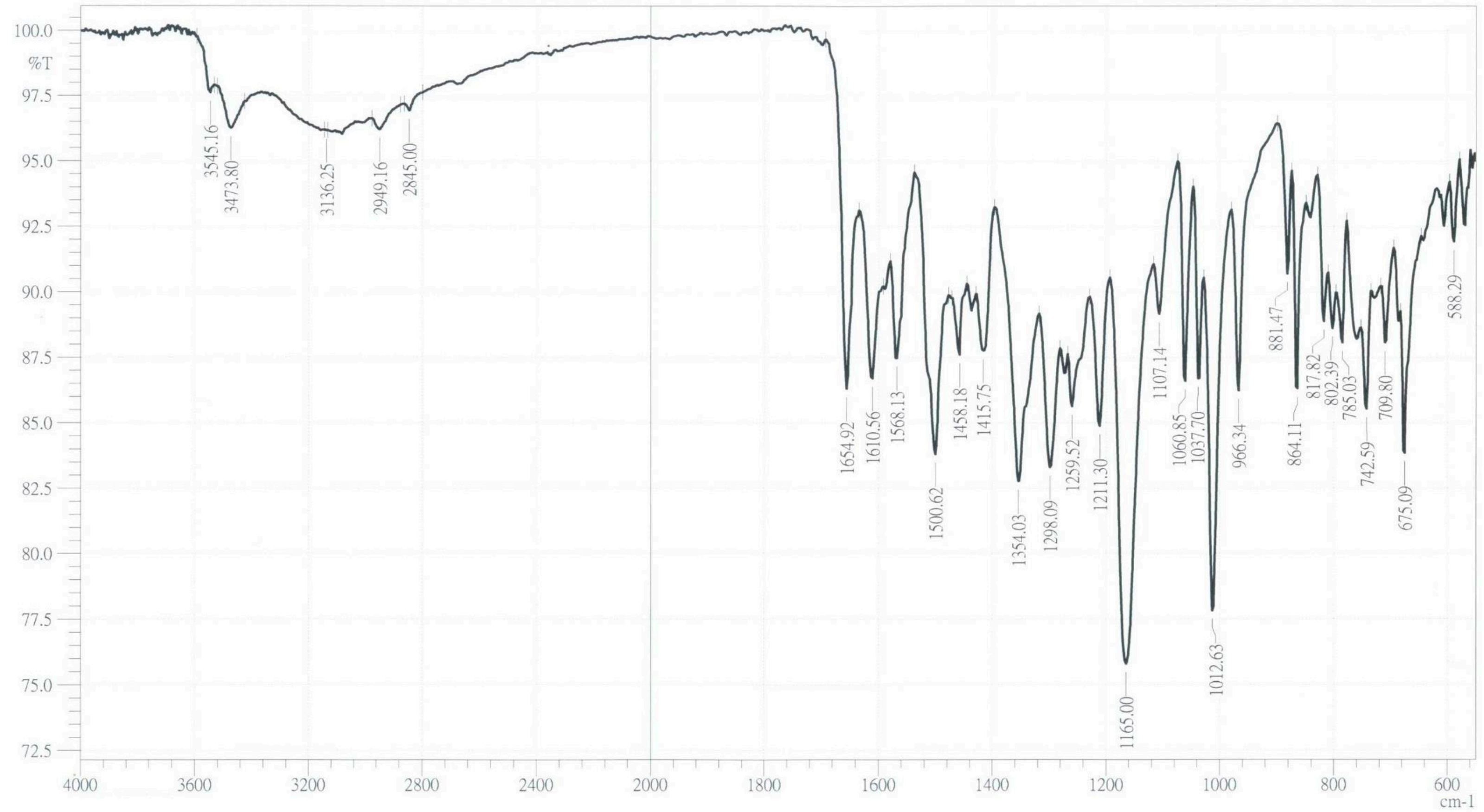

Figure S14. IR spectrum of **FL7**.
